# MicroRNAs induced in melanoma treated with combination targeted therapy of Temsirolimus and Bevacizumab

**DOI:** 10.1186/1479-5876-11-218

**Published:** 2013-09-18

**Authors:** Aubrey G Wagenseller, Amber Shada, Kevin M D’Auria, Cheryl Murphy, Dandan Sun, Kerrington R Molhoek, Jason A Papin, Anindya Dutta, Craig L Slingluff Jr

**Affiliations:** 1 Department of Surgery, Division of Surgical Oncology, University of Virginia School of Medicine, Charlottesville, VA, USA; 2 Department of Biomedical Engineering, University of Virginia School of Medicine, Charlottesville, VA, USA; 3 Department of Biochemistry and Molecular Genetics, University of Virginia School of Medicine, Charlottesville, VA, USA

## Abstract

**Background:**

Targeted therapies directed at commonly overexpressed pathways in melanoma have clinical activity in numerous trials. Little is known about how these therapies influence microRNA (miRNA) expression, particularly with combination regimens. Knowledge of miRNAs altered with treatment may contribute to understanding mechanisms of therapeutic effects, as well as mechanisms of tumor escape from therapy. We analyzed miRNA expression in metastatic melanoma tissue samples treated with a novel combination regimen of Temsirolimus and Bevacizumab. Given the preliminary clinical activity observed with this combination regimen, we hypothesized that we would see significant changes in miRNA expression with combination treatment.

**Methods:**

Using microarray analysis we analyzed miRNA expression levels in melanoma samples from a Cancer Therapy Evaluation Program-sponsored phase II trial of combination Temsirolimus and Bevacizumab in advanced melanoma, which elicited clinical benefit in a subset of patients. Pre-treatment and post-treatment miRNA levels were compared using paired t-tests between sample groups (patients), using a p-value < 0.01 for significance.

**Results:**

microRNA expression remained unchanged with Temsirolimus alone; however, expression of 15 microRNAs was significantly upregulated (1.4 to 2.5-fold) with combination treatment, compared to pre-treatment levels. Interestingly, twelve of these fifteen miRNAs possess tumor suppressor capabilities. We identified 15 putative oncogenes as potential targets of the 12 tumor suppressor miRNAs, based on published experimental evidence. For 15 of 25 miRNA-target mRNA pairings, changes in gene expression from pre-treatment to post-combination treatment samples were inversely correlated with changes in miRNA expression, supporting a functional effect of those miRNA changes. Clustering analyses based on selected miRNAs suggest preliminary signatures characteristic of clinical response to combination treatment and of tumor BRAF mutational status.

**Conclusions:**

To our knowledge, this is the first study analyzing miRNA expression in pre-treatment and post-treatment human metastatic melanoma tissue samples. This preliminary investigation suggests miRNAs that may be involved in the mechanism of action of combination Temsirolimus and Bevacizumab in metastatic melanoma, possibly through inhibition of oncogenic pathways, and provides the preliminary basis for further functional studies of these miRNAs.

## Background

Targeted therapies directed at commonly overexpressed pathways in melanoma have induced clinical responses. The BRAF inhibitor vemurafenib was recently approved by the FDA for BRAF-mutant metastatic melanomas [1]. However, the response duration is short, and patients with wild-type BRAF (BRAF^WT^) do not benefit. Many other single-agent regimens have failed to achieve lasting cures in melanoma patients, perhaps because of parallel and redundant cell survival signaling pathways [2]. Thus, there is a need to target multiple pathways.

The PI3K-AKT-mTOR pathway is constitutively activated in many melanomas, leading to increased cell growth, proliferation, and survival [3,4], and mTOR inhibition with Temsirolimus or sirolimus [rapamycin] has antitumor activity in preclinical models of melanoma [5,6]. However, in a phase II trial of single agent Temsirolimus in patients with advanced melanoma, the overall response rate was only 3% (1/32) [7].

The BRAF^V600E^ mutation provides constitutive activation of the MAPK pathway, making it independent of upstream growth factor signaling; however, melanomas with a driver mutation other than the BRAF mutation may be more dependent on growth factors and upstream signaling. We have found that IGF-1, bFGF, HGF and vascular endothelial growth factor (VEGF) serve both autocrine and paracrine functions, to support melanoma cell proliferation and migration [8] [Shada et al. manuscript in preparation]. VEGF blockade is of particular interest because of its antiangiogenic effects, but also because of the role of VEGF in autocrine growth stimulation of VEGFR2^+^ melanomas [6,8,9]. Single agent therapy with Bevacizumab has had variable results, with response rates of 0% (0/16) and 17% (6/35) in two studies [10,11]. However, our laboratory identified synergistic anti-tumor activity in vitro with combination mTOR inhibition and VEGF blockade [6]. Additional synergy may be available in vivo by blocking VEGF-mediated angiogenesis, independent of tumor cell expression of VEGFR2. Thus, we evaluated combination therapy with Temsirolimus and Bevacizumab in advanced melanoma in a Cancer Therapy Evaluation Program (CTEP)-sponsored phase II clinical trial (NCT00397982). Clinical activity, with objective responses by RECIST (Response Evaluation Criteria in Solid Tumors), was demonstrated in that study [12]. Correlative studies of molecular effect of this combination therapy included analysis of miRNA expression changes with treatment, which is the focus of the present report.

miRNAs are non-coding RNAs consisting of 17–25 nucleotides that regulate protein expression by directly binding and negatively regulating messenger RNAs, by either translational inhibition or degradation [13]. They are implicated in nearly all cellular processes, including cell growth, apoptosis, differentiation, proliferation and invasion/metastasis [13-15]. A growing body of evidence indicates that miRNAs are deregulated in cancer: miRNAs that bind tumor suppressors are often overexpressed, and those that bind oncogenes are under expressed (reviewed in [13], examples in [16-18]). miRNA expression profiling holds promise for predicting and monitoring therapeutic response to targeted therapies [19]. However, little is known about how targeted therapies impact miRNA expression in melanoma, and there are limited data on miRNA expression in vivo in melanoma metastases [20]. We are unaware of prior reports of miRNA profiling of melanoma metastases after mTOR or VEGF inhibition. A more intimate knowledge of the effect of targeted therapies on miRNA expression will help to identify miRNAs involved in targeted drug pathways and, ultimately, to suggest how miRNA expression changes may guide therapy decisions.

We have investigated miRNA expression in metastatic melanoma tissue samples treated with combination Temsirolimus and Bevacizumab. Samples were obtained prior to treatment, after Temsirolimus alone, and after combination treatment. We identified the most significantly altered miRNAs and conducted a preliminary investigation of the significance of these alterations for the action of combination Temsirolimus and Bevacizumab therapy in melanoma.

## Methods

### Clinical study

From 5/8/2007 to 2/8/2011, 17 patients with stage III or IV melanoma were enrolled in a CTEP-sponsored phase II clinical trial of combination Temsirolimus and Bevacizumab. Tumor was accessible for biopsy in 13 patients; for 12 of these, tumor samples were evaluated for miRNA expression by Exiqon’s 6th generation microRNA Array (see Additional file 1: Table S1). Patients were assessed every 8 weeks, using clinical staging (CT scans, MRI, physical exam). Clinical tumor responses were measured using RECIST criteria modified to account for tumor biopsies. Tumor biopsies were obtained at study entry on day 1 (Cycle 1, Day 1), day 2 (Cycle 1, Day 2, 24 h after treatment with Temsirolimus alone), and day 23 (Cycle 2, Day 9, after treatment with both Temsirolimus and Bevacizumab). All of the research involving human subjects was approved by the University of Virginia’s IRB (Human Investigation Committee, HIC 5202, 10598, and 12471), in accordance with assurances filed with and approved by the Department of Health and Human Services.

### Cells and tissues

Cell lines were cultured from tumor-involved lymph nodes resected from patients at the University of Virginia (VMM18, VMM39) or Duke University (DM13, DM122), as previously described [21-24]. Their BRAF and NRAS mutation status and expression of VEFR2 are included in Additional file 2. Cell lines were cultured in RPMI-1640 (Mediatech, Inc., Manassas, VA) supplemented with 5% fetal bovine serum, 2 mmol/L L-glutamine, penicillin (100 units/mL), and streptomycin (100ug/mL) at 37°C in 5% CO_2_, unless otherwise indicated. Tissue biopsies were prepared immediately upon excision by transfer to Bio Repository and Tissue Research Facility (BTRF) staff directly in the operating room or procedure room. In accord with the protocol, a portion was placed in liquid nitrogen after removal and stored at -80°C, and another portion was formalin-fixed and subsequently paraffin-embedded (FFPE). Additional file 1: Table S1 lists samples available and analyzed for each patient.

### RNA isolation and quality control

For miRNA microarray analysis, RNA was isolated from sections cut from FFPE tissue using the miRNeasy FFPE kit (Qiagen, Valencia, CA). For in vitro microarray validation, total RNA was extracted from cell lines using Qiazol (Qiagen). For mRNA target analysis after combination treatment, 20 samples were evaluated in 10 patients: for 16 samples, frozen tumor pieces were allowed to thaw in RNAlater-ICE (Life Technologies, Grand Island, NY) overnight at -20°C and then were mechanically rendered into powder at -180°C in vapor-phase N_2_. The powder was placed in lysis buffer, and RNA was isolated using the RNeasy Midi Kit for Fibrous Tissue (Qiagen). For the remaining four samples (see Additional file 1: Table S1), extraction was performed with Qiazol crude extraction (Qiagen), followed by cleanup with the RNeasy Mini Kit (Qiagen). For all RNA extractions, concentration and purity were assessed with Nanodrop 8000 technology.

### MicroRNA microarray

Microarray analysis was conducted at Exiqon, using their miRCURY LNA microRNA Array (6th gen) with probe sets for over 1,300 human miRNAs and using the Bioanalyser2100 (Agilent, Santa Clara, CA) and Nanodrop instrument for quality control. Following hybridization, signals were background-corrected and then normalized using the global Lowess regression algorithm. Further details regarding Exiqon’s protocol can be found in supplementary data (see Additional file 2). The data are available in GEO (GSE37131). Unsupervised hierarchical clustering was performed on all samples and on the top 50 miRNAs with the highest standard deviations across the sample set. In addition, aliquots of miRNA extract from 5 samples were resubmitted to Exiqon for analysis, to control for shipping conditions and intraassay variability.

### Data analysis

To compare pre- and post-treatment miRNA levels, paired t-tests were performed between sample groups, using a p-value < 0.01 for significance. A permutation-based statistical test resulted in highly similar ranking of genes, corroborating the results from the t-tests [25]. Delta log median ratios (dLMR) were calculated by subtracting the pre-treatment log median ratio [log2 (Hy3/Hy5)] (LMR; Hy3,5 are fluorescent labels) from the post-treatment LMR.

### In vitro analysis

Bevacizumab (25 mg/mL) was obtained from the University of Virginia Infusion Center and used at 50 ug/mL. Rapamycin (R-500) was purchased from LC Laboratories, and a stock solution was made in dimethyl sulfoxide (DMSO) and used at 10 nmol/L. Melanoma cells were plated on 100 mm plates and allowed to adhere overnight. After 24 h, cells were washed and either harvested (untreated, 0 hour samples), or treated with serum alone, rapamycin, Bevacizumab, or both. Cells were harvested at 24 h or 48 h. RNA was extracted, and qRT-PCR performed as described below. P-values were obtained by a ratio paired t-test.

### Quantitative reverse transcription-PCR (qRT-PCR)

For in vitro analysis, qRT-PCR was performed in triplicate with the TaqMan MicroRNA assays kit (Applied Biosystems, Carlsbad, California), following manufacturer’s directions. The U6 small nuclear RNA, RNU6, was used for normalization (Applied Biosystems). For mRNA target validation, RNA was extracted from eight post-combination treatment tumor samples, and 3–4 micrograms total RNA was reverse-transcribed using High Capacity cDNA Archive kit (Applied Biosystems), followed by qPCR with Power SYBR Green Master Mix (Applied Biosystems) in triplicate. Housekeeping genes used for normalization of mRNA levels included ActB and HPRT1. Primer sequences for ActB, HPRT1 and the 18 target genes are in the supplemental data (see Additional file 2).

### MicroRNA-mRNA correlations

To assess correlations between miRNA changes and proposed target gene expression changes, we assessed fold-induction of the 15 differentially expressed miRNAs (2^dLMR^) and the log-transformed change in gene expression level for each patient: log_10_ (post-treatment normalized expression value divided by pre-treatment value). Plots were constructed for each miRNA-log_10_mRNA pair (25 total). Trend lines were added; correlation coefficients and their significance were calculated using MedCalc software (Mariakerke, Belgium).

### Clustering analyses

To obtain preliminary data on whether pre-treatment miRNA levels or miRNA changes with treatment correlate with clinical outcome, clustering analyses were performed with expression values (log median ratios, LMRs and delta log median ratios, dLMRs) for combinations of miRNAs. Ward’s method was used to hierarchically cluster patients with similar expression signatures. Similarities among miRNA expression profiles of patients were quantified using Pearson correlation coefficients. Three types of analyses were performed: before and after treatment, responders versus non-responders, and BRAF^wt^ versus BRAF^V600E^ tumors. For each analysis, unsupervised clustering was performed using expression values for all miRNAs and a second “semi-supervised” analysis was performed using a subset of miRNAs, selected based on t-test p-value and effect size cut-offs.

## Results

### miRNA expression profiles of melanoma tumor samples from the same patient cluster together, and expression measurements are reproducible

**Figure 1:**
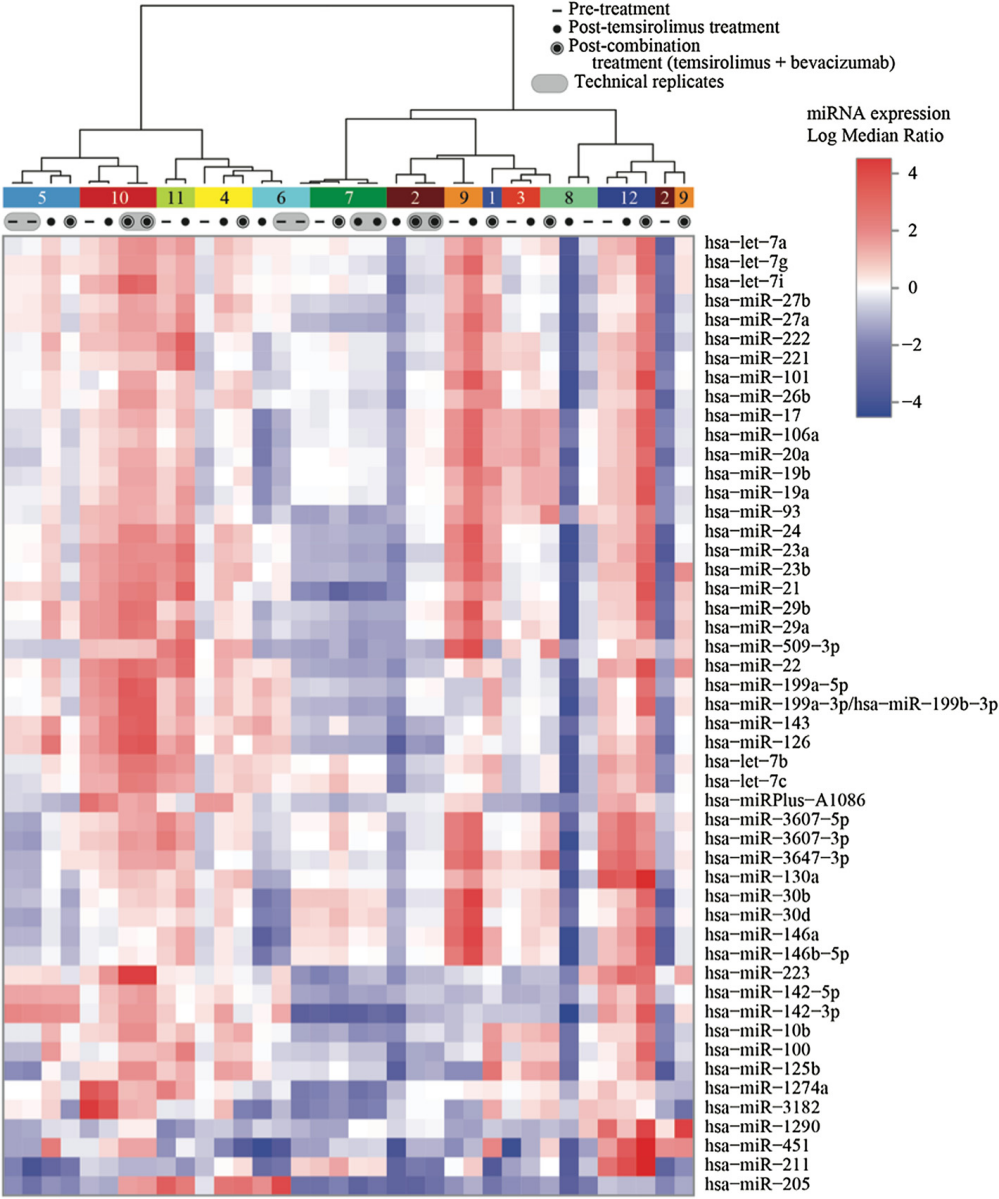
**Unsupervised clustering analysis of miRNA expression.** The heat map illustrates the result of the two-way hierarchical clustering of miRNAs and samples. Each row represents one miRNA and each column represents one sample, including pre-treatment, post-Temsirolimus alone, or post-combination treatment samples from patients #1 through 12. Samples resubmitted for quality assurance purposes are marked by a shaded oval enclosing the duplicate samples. The color scale illustrates the relative expression level (log median ratio, LMR) of a miRNA across all samples: red color represents an expression level above the mean, blue color represents expression lower than the mean. Clustering was performed on all samples and on the 50 miRNAs with the highest standard deviation across the sample set.

To identify changes in miRNA expression with treatment, 31 tumor samples were evaluated for over 1,300 miRNAs using microarray analysis. miRNA was extracted from tumor biopsies: (i) pre-treatment (n = 11), (ii) 24 h after Temsirolimus alone (n = 11), and (iii) after combination therapy with Temsirolimus and Bevacizumab, day 23 (n = 9, Additional file 1: Table S1). The heat map (Figure 1) depicts relative expression levels (log median ratios, LMR) of the 50 miRNAs whose values varied most over the sample set, based on standard deviation. miRNA expression among different patients varies more than expression among different tumor samples from an individual patient. Samples resubmitted for quality assurance clustered with the corresponding originally submitted samples, supporting the reproducibility of the data.

### miRNA expression changes with Temsirolimus alone

**Figure 2:**
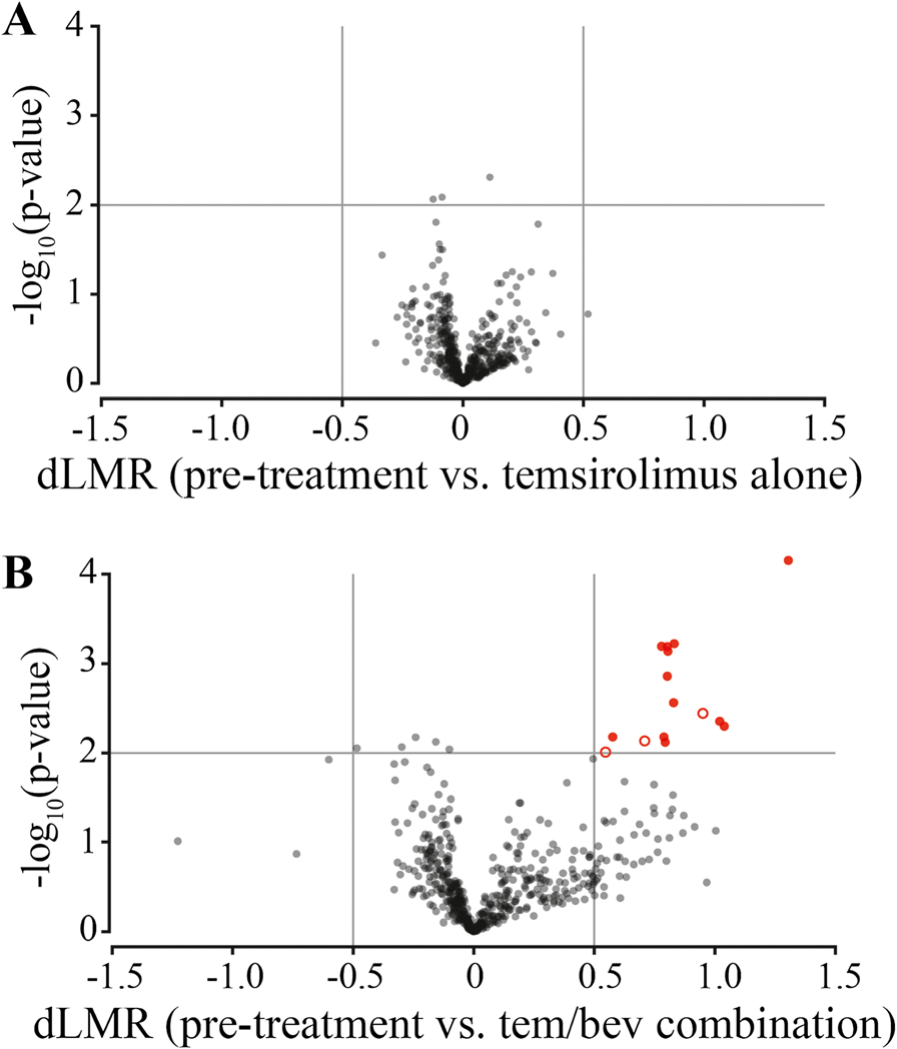
**Significant differential expression of miRNA with treatment.** Volcano plots were generated to facilitate identification of significantly differentially expressed miRNAs with Temsirolimus alone **(A)** and with combination treatment **(B)**. The plot shows fold-change (dLMR) on the x-axis and –log10 (p-value) on the y-axis. Criteria used to identify significantly differentially expressed miRNAs included 1) two-tailed t-test p-value < 0.01 and 2) absolute delta-log median ratio (dLMR) value > 0.5. miRNA marked by solid red circles are putative tumor suppressors.

To identify miRNAs significantly altered by Temsirolimus, we compared miRNA expression levels after Temsirolimus alone to pre-treatment levels. miRNA had to meet two criteria to be considered significantly altered: 1) two-tailed t-test p-value < 0.01, and 2) absolute difference between normalized expression values (delta-log median ratio, dLMR) > 0.5. Three miRNAs (miR-2115, -488, -2116) were significantly differentially expressed after treatment with Temsirolimus alone; however, none met the second criterion (Figure 2A). miR-100, known to target mTOR [26], had a dLMR > 0.5, but was not significantly different in the two-tailed t-test.

### 15 miRNAs are differentially expressed in melanoma tumor samples following combination treatment with Temsirolimus and Bevacizumab

Using the same methods and criteria to identify miRNAs significantly altered with Temsirolimus alone, we identified 15 miRNAs significantly differentially expressed in melanoma tumor samples following combination treatment compared to pre-treatment (Table 1). The same 15 miRNAs, plus the hsa-miRPlus-A1086, were identified when using a false discovery rate cut-off of 13% from a permutation-based statistical test (instead of p < 0.05 using a t-test). All fifteen were significantly upregulated and were increased by 1.4- to 2.5-fold compared to pre-treatment levels (Figure 2B). Twelve of these 15 miRNAs (Table 1, Figure 2B) possess tumor suppressor functions in various cancer types (citations in Table 1), including melanoma.

**Table 1:** miRNAs differentially expressed after combination treatment with Temsirolimus and Bevacizumab

**miRNA**	**dLMR**	**Fold-change**	**p-value (-log10)**	**Disease**	Target*
**125b**	1.30	2.46	7.37E-05 (4.13)	Melanoma [27,28]	AKT^2^
				Hepatocellular cancer [29]	LIN28B^1,2,3^
**320a**	0.83	1.78	6.10E-04 (3.21)	miR-320 family: Insulin-resistant adipocytes [30], Diabetic myocardial microvascular endothelial cells [31], Murine bronchial epithelial cells treated with benzo[a]pyrene [32], TargetScan	PI3-K^2^
**320b**	0.81	1.75	7.28E-04 (3.14)		CDK6^2^
**320c**	0.80	1.74	6.65E-04 (3.18)		
**320d**	0.78	1.72	6.32E-04 (3.20)		
**320e**	0.80	1.74	1.36E-03 (2.87)		
**let-7 family**				Lung cancer [33]	HMGA2^1,2,3^
				Lung cancer [16]	RAS^1,2,3^
				Hepatocellular cancer [34]	LIN28B^1,2^
**7b**	0.79	1.73	6.40E-03 (2.19)	Melanoma [35]	CCND1^1,2^
**7c**	0.83	1.78	2.75E-03 (2.56)		
10b	0.95	1.93	3.65E-03 (2.44)		
**29c**	1.02	2.03	4.45E-03 (2.35)	Melanoma [36]	DNMT3A/B^2^
				Hepatocellular cancer [37]	BCL2, MCL1^1,2^
				Cervical cancer (HeLa) [38]	PI3-K^1,2^
				Cervical cancer (HeLa) [39]	MYBL2^1,2,3^
				Human solid tumors [40]	
**100**	1.04	2.06	5.05E-03 (2.30)	Human CMV [26]	mTOR^1,2^
				Clear cell ovarian cancer [41]	mTOR^2,3^
				Prostate [42]	mTOR^1,2^
					SMARCA5^1,2^
					SMARCD1^1,2^
**145**	0.57	1.48	6.61E-03 (2.18)	NSCLC [17]	C-MYC^1,2^
				Colon, breast cancer [43,44]	
140-3p	0.71	1.64	7.28E-03 (2.14)		
**99a**	0.79	1.73	7.59E-03 (2.12)	Prostate [42]	mTOR ^1,2^
					SMARCA5 ^1,2^
					SMARCD1^1,2^
4328	0.55	1.46	9.80E-03 (2.01)		

miRNAs in bold are putative tumor suppressors; citations pertain to both the disease and associated target. *Methods used for target validation are noted with superscript as follows: 1, 3’UTR luciferase reporter assay; 2, inverse correlation of miRNA and target protein levels; 3, inverse correlation of miRNA and target mRNA levels.

### In vitro analysis

To determine the extent to which the observed alterations in miRNA expression may be explained by induction of the miRNAs in melanoma cells themselves, expression of the 15 significant miRNAs was measured by qRT-PCR in four melanoma cell lines after culturing with media alone, rapamycin (Temsirolimus analogue), Bevacizumab, or combination of rapamycin and Bevacizumab. qRT-PCR was chosen over microarray analysis for the superior sensitivity, accuracy, and higher dynamic range of qRT-PCR. We first tested five miRNAs (miR-125b, *let-7c*, -29c, -100, -99a) at 24 h and 48 h and found that all were upregulated at least 2-fold with combination treatment after 24 h, 48 h or both (except one miRNA in one cell line: *let-7c* in VMM39; Additional file 3: Figure S1). Less upregulation was observed with rapamycin. Bevacizumab alone had minimal effect except for one VEGFR2^+^ line. The effect of combination treatment was more than additive.

We then tested the remaining 10 miRNAs at 48 h. For 3 cell lines (VMM18, VMM39, DM122), there was at least a 2-fold upregulation with combination treatment for 5, 9, and 1 of the miRNAs, respectively (Additional file 4: Figure S2). Among these, most striking were increases of *let-7b* for VMM18 and VMM39 (28 and 18-fold, respectively). In all cases with at least 2-fold upregulation, combination treatment induced greater upregulation than either agent alone.

### Target identification for the significant tumor suppressor miRNAs

To explore further the mechanism by which combined Temsirolimus and Bevacizumab may elicit tumor control, we sought potential oncogenic targets of the 12 tumor suppressor miRNAs (Figure 2B) identified in the microarray analysis—targets whose altered expression was likely to have a functional effect relevant to melanoma and/or to the treatments used in this study. We used the computational target prediction program TargetScan and published experimental evidence of miRNA-target interactions to identify potential targets. Among the numerous genes identified, we chose to focus on 15 targets likely to play a role in melanoma and in tumorigenesis generally, relying primarily on published evidence of a potential miRNA-mRNA interaction (Table 1). The sources cited in Table 1 include two types of evidence: the 3’UTR luciferase reporter assay supports a direct interaction between a miRNA and its mRNA target, where an inverse relationship between miRNA and target protein or mRNA levels is indirect evidence of a relationship.

### Pilot exploration of selected miRNA-target interactions

**Figure 3:**
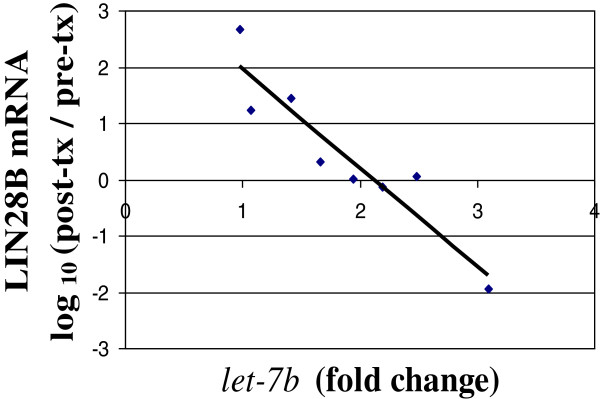
**Inverse correlation between changes in miR-let-7b and proposed target LIN28B mRNA.** miR-let-7b fold change (2^dLMR) with combination treatment is plotted against log_10_ of the percent change in LIN28B mRNA with combination treatment (post-treatment expression/pre-treatment expression) for each patient for whom both pre-treatment and post-combination treatment samples were available. The slope and intercept of the linear trendline were -1.75 and 3.7, respectively.

To conduct a preliminary analysis of relationships between the 12 tumor suppressor miRNAs and their selected targets with establish roles as oncogenes in melanoma samples, we measured messenger RNA by qRT-PCR for the 15 target genes in pre- and post-combination treatment samples. To assess associations between changes in miRNA and mRNA in each patient, we plotted the miRNA fold-induction with combination treatment against the corresponding log_10_ (fold change in target gene expression level) for each patient, for all 25 miRNA-oncogene pairings. There were inverse correlations for 15 of the 25 pairings (see Additional file 5: Table S2), inverse relationships are expected if these miRNA inhibit their proposed targets in melanoma. Of these 15 inverse correlations, 2 had a significant p-value: miR-let-7b and LIN28B (p = 0.0008, Figure 3) and miR-let-7c and LIN28B (p = 0.0012). For the remaining miRNA-target comparisons, it is yet to be determined whether the lack of significant inverse correlations implies that these genes are not targeted by the proposed miRNAs in melanoma cells or whether they are regulated by other post-transcriptional processes that complicate the expected inverse relationship.

### Association between miRNA expression profiles and clinical response

**Figure 4:**
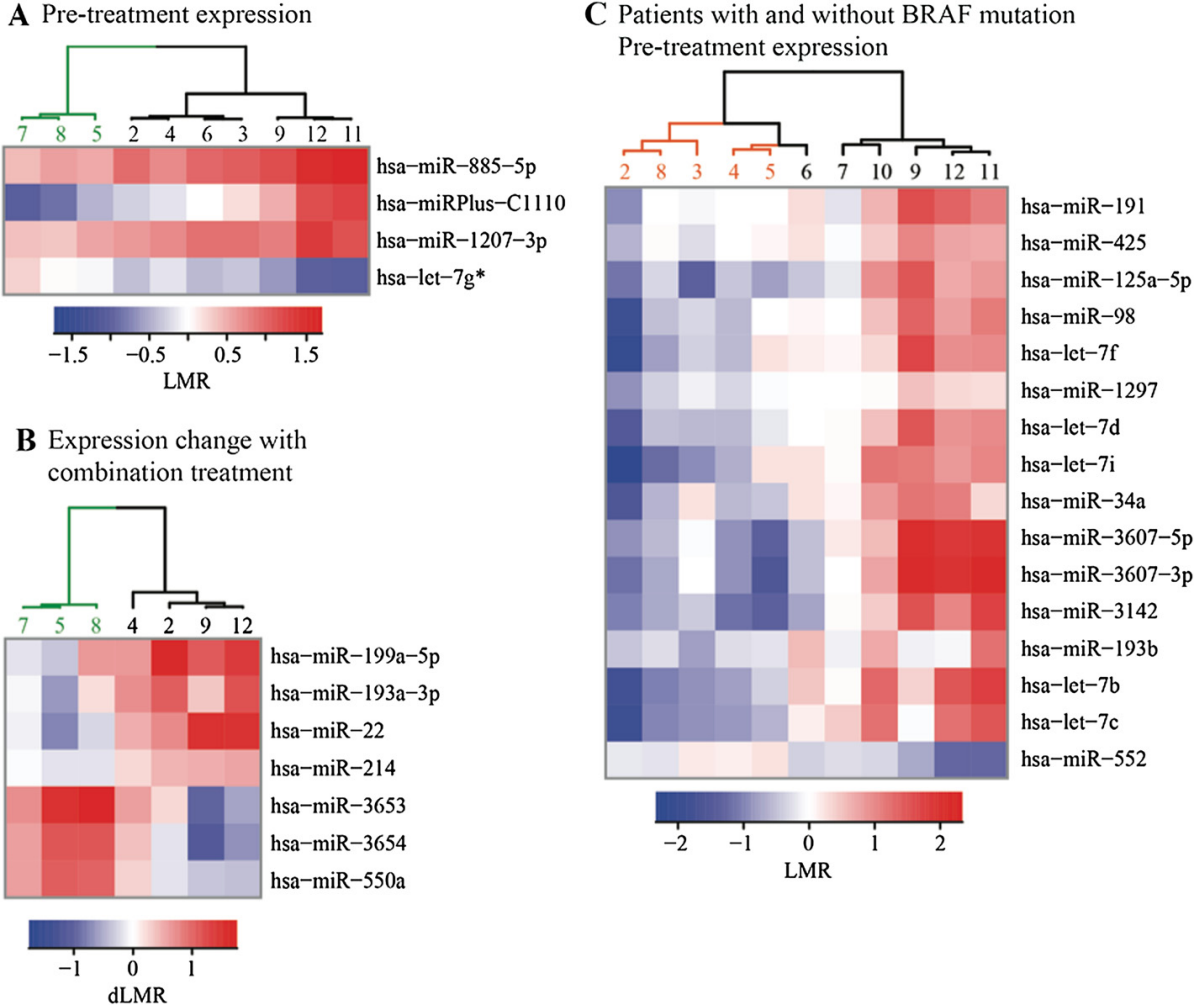
**Clustering analysis of miRNA expression according to clinical outcome and BRAF tumor status.** The heat map illustrates the result of the two-way hierarchical clustering of pre-selected miRNAs and samples. The color scale illustrates the relative expression level or changes in expression level across all samples: red color represents an expression level above the mean and blue color represents expression lower than the mean. Patients #5, 7, and 8 had progressive disease (PD), patient #6 and 9 had partial responses (PR), and the remaining patients had stable disease (SD). **(A)** Clustering was performed with pre-treatment expression values (LMRs) for miRNAs with t-test p-value < 0.01 and effect size > 0.5. **(B)** Clustering was performed with changes in miRNA expression (dLMRs) with combination treatment for miRNAs whose t-test p-value < 0.04 and effect size > 0.5. **(C)** Clustering was performed with pre-treatment expression values (LMRs) for miRNAs whose t-test p-value < 0.02 and effect size > 0.5, using p-values obtained from two-tailed t-tests comparing the average expression level for patients with BRAF mutant melanomas (in orange) with those who had wild-type BRAF melanomas.

We conducted a preliminary investigation to explore whether miRNA expression profiles pre- or post- treatment may be associated with clinical outcome. Using both unsupervised and supervised clustering analyses, we evaluated whether those with treatment failure (PD, progressive disease) might be distinguished from those with stable disease (SD) or partial responses (PR) (see Additional file 1: Table S1 for clinical outcome for each patient, manuscript submitted). Clustering analyses were performed using expression values of miRNAs selected for consistent and significant alterations in patients with SD or PR, compared to those with PD. We also performed clustering analyses comparing pre- and post-treatment miRNA expression (based on LMR values). A total of 30 clustering analyses were performed, four of which resulted in clustering of patients with PD (#5, 7, and 8) separately from those with SD or PR: one based on pre-treatment LMRs (Figure 4A), one on post-combination treatment LMRs (not shown), one on post-combination treatment dLMR values (Figure 4B) and one on post-Temsirolimus dLMR values (not shown). miR-193a-3p and -199a-5p are included in the post-combination treatment dLMR signature (Figure 4B) and are upregulated to a greater degree in responders compared to non-responders.

We also assessed whether a miRNA signature might distinguish patients based on BRAF mutation status. Pre-treatment miRNA expression (LMR) differed for BRAF^wt^ and BRAF^V600E^, with 16 miRNAs in that preliminary signature (Figure 4C). Five of them are in the let-7 family, all upregulated in BRAF^WT^ melanomas. An additional analysis based on post-combination treatment dLMRs also resulted in clustering of patients according to BRAF tumor status (not shown).

## Discussion

In this preliminary investigation of miRNA expression in human melanoma tissue treated with targeted therapies, we report significant upregulation of 15 miRNAs in metastases following combination targeted therapy with the mTOR inhibitor Temsirolimus and the anti-VEGF antibody Bevacizumab for advanced melanoma. Remarkably, the observed change was upregulation with treatment, for all 15. Twelve of the 15 have tumor suppressor activities in melanoma or other cancers (Table 1). Because this regimen has clinical activity, it is possible that these altered miRNAs may have a role in that activity.

It is also interesting that no miRNAs were significantly altered 24 hours after treatment with Temsirolimus alone, despite the critical nature of the signaling pathway targeted by Temsirolimus. Rapamycin has been shown to modulate miR-1 expression; however, this relationship was identified in differentiating myoblasts and in mouse regenerating skeletal muscle, not in human melanoma cells. More importantly, mTOR’s influence over miR-1 expression was mediated through MyoD, a transcription factor specific for skeletal myogenesis [45]. Since miRNA expression depends on intrinsic cellular factors, this relationship is unlikely to be found in human melanoma cells. Importantly, the lack of change observed with mTOR inhibition alone is consistent with the lack of clinical activity seen with Temsirolimus alone in metastatic melanoma [7] and may provide some insight into the lack of clinical impact with this agent alone. It is possible that treatment with Temsirolimus alone for greater than 24 hours would alter miRNA expression profiles more significantly. However, we would expect some changes within 24 hours, especially since we have observed consistent decreases in phospho-S6Kinase in these metastases 24 h after Temsirolimus therapy [12].

We did not test the effects of Bevacizumab alone in the trial; so, it is possible that the significant alteration of miRNA levels seen with combination treatment is due to Bevacizumab alone rather than the combination. However, the in vitro analysis revealed minimal effect of Bevacizumab alone on miRNA expression in most of the 4 tested melanoma cell lines. In addition, single agent therapy with Bevacizumab has had variable results in melanoma patients, with response rates of 0% (0/16) and 17% (6/35) in two studies [10,11]. mTOR is important in cell survival during stress, and VEGF blockade can induce hypoxic stress. Thus, there is rationale for the combination effect to exceed the effect of either agent alone, and this is consistent with the synergistic anti-tumor activity we have observed in vitro [6]. Future studies may clarify the mechanism of synergy of this combination therapy.

To obtain preliminary data on whether miRNA changes observed in the tumors may be explained by direct effects on melanoma cells themselves, we analyzed the effect of either one or both agents on miRNA expression in human melanoma cell lines. These data reveal the heterogeneity of individual melanomas. However, striking and global increases in almost all 15 miRNAs are induced by combination treatment in the VEGFR2^+^ melanoma VMM18, where VEGF can have a direct effect on the melanoma cells themselves [6], with more transient effects for DM13 (also VEGFR2^+^). In the VEGFR2^neg^ lines, VMM39 and DM122, upregulation of miRNAs with combination treatment may be explained by blockade of direct effects of VEGF on VEGFR3, which is widely expressed on human melanomas and is phosphorylated in both of these cell lines [8]. Thus, by combined effect of mTOR inhibition and VEGF blockade on VEGFR2 and VEGFR3 signaling, the effect of this combination therapy may be explained in part by direct effects of both agents on melanoma cells. However, some observed changes in miRNA expression in biopsies are likely due to other cells in the tumor microenvironment as well.

miRNA expression is mediated through strict regulation of both transcription and post-transcriptional maturation [46]. The targeted therapies used in this study may target those processes directly or indirectly. Numerous drugs alter miRNA expression in cancer, including cisplatin and 5-fluorouracil in esophageal cancer [47] and 1α,25 dihydroxyvitamin D3 and testosterone in prostate cancer [48]. MiR-320a and miR-29a/b were upregulated with treatment in those studies, respectively, which was also observed in the present study. It is possible that combination Temsirolimus and Bevacizumab similarly directly induces miRNA expression. Alternatively, upregulation may represent a broad molecular response to treatment, downstream of the anti-tumor activity of the drugs. Other potential regulators of miRNA expression include the miRNA targets themselves. For example, both MYC and LIN28B negatively regulate *let-7* expression at the level of transcription and processing, respectively [49,50]. Such auto-regulatory loops likely account for the lack of precise linear inverse correlations observed with analysis of miRNA and target mRNA expression (see Additional file 5: Table S2).

A potential mechanism by which upregulation of these miRNAs may exert an anti-tumor effect involves the influence of miR-125b and miR-100 over the Akt/mTOR pathway. miR-125b was the miRNA most upregulated with combination treatment in this study (Table 1). It is a putative tumor suppressor in melanoma [27,29], and its expression is lower in metastasizing vs. non-metastasizing melanoma [27]. Overexpression of miR-125b can produce senescence in melanoma cells [28]. A potential target of miR-125b is Akt3, which is overactive in melanoma and whose expression increases during melanoma progression [51]. Downregulation of miR-125b may contribute to progression of melanoma via Akt3 upregulation [28]. Thus, upregulation of miR-125b may contribute to melanoma regression. miR-100 was also upregulated with combination treatment. It targets mTOR and the mTOR-associated protein raptor [26,42]. Overexpression of miR-100 enhances in vitro sensitivity to rapamycin in ovarian cancer cell lines [41]. The observed upregulation of miR-125b and miR-100 with combination treatment may reflect additive or synergistic inhibition of the Akt3/mTOR pathway with combination treatment, mediated by three mechanisms: direct inhibition of mTOR by Temsirolimus, translational inhibition of mTOR by upregulation of miR-100, and inhibition of the Akt3 pathway by upregulation of miR-125b.

Another putative tumor suppressor among the 15 miRNAs is the *let-7* family. *Let-7b* is significantly downregulated in primary melanomas compared to benign nevi, inhibits cyclin D1 in melanoma cells, and inhibits cell cycle progression and anchorage-independent growth when overexpressed in melanoma cells [35]. Furthermore, the let-7 family (all with a similar sequence necessary for target recognition) suppresses the oncogene HMGA2 [33]. The effects we observed in melanoma cells are most striking for *let 7b*, and its strong inverse correlation with LIN28B expression supports further investigation of this miRNA-mRNA pair as a possible mediator of therapeutic effects of this combination therapy. Definitive association of let7b and LIN28B require luciferase reporter assays; such studies have performed for human hepatocellular cancer and confirm the role of let7b as a negative regulator of LIN28B [34].

Results of the clustering analyses (Figure 4) suggest other miRNAs, such as miR-193a-3p and -199a-5p (Figure 4B) that may also be worth investigating as possible molecular markers of treatment response. miR-193a was found to function as a tumor suppressor in several cancer types [52] and is under expressed in melanomas containing a BRAF mutation [53]. miR-199a-5p and -199a-3p are both processed from pre-miR-199a, whose promoter region is important for expression of both miRNAs [54]. miR-199a-3p targets mTOR and c-Met in its role as a tumor suppressor in hepatocellular carcinoma and enhances susceptibility to hypoxia when its levels are restored [55]. Thus, it is interesting that miR-199a is upregulated in responders compared to non-responders, with a combination therapy that is presumed to act in part through hypoxia-induced cell death (Bevacizumab). A larger clinical study is needed to validate whether miRNAs within these signatures may predict treatment response; however, we provide a foundation for future development of a prognostic model.

A limitation of this study is the modest number of patients enrolled and studied, which was constrained by the sample size of the phase II clinical trial [12]. The accrual goal was 20; the actual accrual was slightly lower, at 17. Three of 17 were taken off study drugs before day 23. Overall 8 patients had biopsies at all three time point (days 1, 2, 23) – this is a small number, limited by realities of the accessibility of tumor for biopsy and the requirements to manage patient safety in accord with the protocol. However, there is substantial statistical power in the analysis because these were sequential biopsies from the same patient in each of those cases. Studies with similar and smaller sample size have also been informative for miRNA studies of human tissues [56,57]; however, it will be valuable to test these findings in a larger dataset when available. Nonetheless, the study represents, to our knowledge, the first study of miRNA expression in melanoma metastases before and after combination targeted therapy, and one of few that evaluates tumor on repeat biopsies.

Another limitation of this study is the fact that we did not analyze the expression of all potential targets of the 12 tumor suppressor miRNAs in the treatment samples. This was beyond the scope of the present study, and instead we focused on targets likely to have a functional effect relevant to melanoma and/or the treatments used in this study. Future studies expanding on these findings may reveal other targets with functional significance with regards to upregulation of these 15 miRNAs. Furthermore, the RNA extracts used in the target analysis were prepared using two different methods. Thus, the preliminary target validations are acknowledged to be just a pilot data set. Finally, we collected both frozen tissue and FFPE tissue from these patients, but we intentionally did the work on FFPE samples because they will have broad relevance for studies involving archival specimens, and because of published work that validates the accuracy of miRNA expression in FFPE samples [58-60].

We report preliminary results that establish the basis for further expansion. In the future, an independent and larger set of samples should be used to validate these preliminary results. After validation of these findings, further functional studies are needed to determine the mechanism of induction of these miRNAs and their role in the mechanism of action of combination Temsirolimus and Bevacizumab.

## Conclusions

In summary, we report significant changes in miRNA expression in a cohort of patients after treatment with a novel combination regimen in metastatic melanoma that has had encouraging clinical activity. Treatment with Temsirolimus alone failed to elicit any significant changes in miRNA expression, whereas combination treatment with Temsirolimus and Bevacizumab results in distinctly different miRNA expression profiles, emphasizing the enhanced efficacy of combination therapy compared to single-agent treatment. Twelve of the fifteen miRNAs significantly upregulated with combination treatment possess tumor suppressor properties, and thus, this study suggests miRNAs for further functional study that may be involved in the mechanism of action and clinical activity of combined mTOR and VEGF inhibition. Overall, this study addresses the need for further in vivo studies of miRNA expression in melanoma and takes preliminary steps toward incorporating miRNA expression profiling into melanoma therapeutics by illuminating how targeted therapies impact miRNA expression in melanoma. Thus, this study provides further support for the potential of miRNAs to inform clinical decisions by sub-classifying patients susceptible to novel targeted therapies [20].

## Competing interests

None of the authors have any competing interests related to the work in this manuscript.

## Authors’ contributions

AW analyzed the microarray results, performed the in vitro work and target analysis, and drafted the manuscript. AS coordinated the microarray analysis and design of the study. KD and JP performed all statistical analyses, including the clustering analyses. CM assisted with the in vitro work and target analysis. DS assisted with the qRT-PCR analysis. KD participated in the design of the study. AD participated in design of the study. CLS was principal investigator on the clinical trial and conceived of the study, participated in its design and conception, and helped to draft the manuscript. AW, AS, AD, and CLS contributed most to writing and editing the manuscript; all authors have at least reviewed the final manuscript. All authors read and approved the final manuscript.

## Supplementary Material

Additional file 1: Table S1 List of patients and their clinical outcomes and samples analyzed. Click here for file

Additional file 2 **Methods.** Additional details of miRNA microarray analysis and qRT-PCR. Click here for file

Additional file 3: Figure S1 In vitro analysis: Part I. Expression of the first set of miRNAs (miR-125b, *let-7c*, -29c, -100, -99a) in four melanoma cell lines after culturing with media alone, rapamycin (Temsirolimus analogue), Bevacizumab, or combination of rapamycin and Bevacizumab. Click here for file

Additional file 4: Figure S2 In vitro analysis: Part II. Analysis of the remaining 10 miRNAs. Click here for file

Additional file 5: Table S2 Correlation between changes in miRNA and mRNA with combination treatment: correlation coefficients and *p*-values. Negative correlations and significant p-values are in bold. Click here for file
