# Supplementary material for: MicroRNAs induced in melanoma treated with combination targeted therapy of Temsirolimus and Bevacizumab
Source: J Transl Med. 2013 Sep 18;11:218. doi: 10.1186/1479-5876-11-218 (PMC3853033; doi:10.1186/1479-5876-11-218)
Supplement: Additional file 1: Table S1 — List of patients and their clinical outcomes and samples analyzed. [file 1479-5876-11-218-S1.doc]

**Table S1.** Patients and available tissue samples.

| Patient # | Treated | Tumor Tissue Collected | | | Exiqon  analysis | Target Analysis | Outcome |
| --- | --- | --- | --- | --- | --- | --- | --- |
|  |  | Pre-tx | Post-tem | Post-combo |  |  |  |
| 1 | + | +^1^ | +^1^ | +^2^ | c | p^3^, c | SD |
| 2 | + | + | + | + | p, t, c | p, c | SD |
| 3 | + | + | + | ND | p, t | p, c | SD |
| 4 | + | + | + | + | p, t, c | p, c | SD |
| 5 | + | + | + | + | p, t, c | p, c | PD |
| 6 | + | + | + | ND | p, t | ND | PR |
| 7 | + | + | + | + | p, t, c | p, c^3^ | PD |
| 8 | + | + | + | + | p, t, c | p, c^3^ | PD |
| 9 | + | + | + | + | p, t, c | p, c | PR |
| 10^4^ | + | + | + | + | p, t, c | p, c | **NE** |
| 11 | + | + | + | ND | p, t | ND | SD |
| 12 | + | + | + | + | p, t, c | p, c^3^ | SD |
| **Total** | **12**^5^ | **12** | **12** | **9** | **31** | **20** | 33 |
| Key: temsirolimus (tem), combination therapy (combo); pre-treatment (p), post-temsirolimus (t), post-combination therapy (c); partial response (PR), stable disease (SD), progressive disease (PD), and not evaluable (NE); not done (ND).  ^1^ Quantity not sufficient for submission to Exiqon.  ^2^ Not included in Fig.4b clustering analysis because this patient did not have pre-treatment data necessary for calculation of dLMRs.  ^3^ RNA extraction for target analysis was performed with Qiazol crude extraction.  ^4^ Patient’s samples were not included in clinical outcome clustering analyses due to non-evaluable (NE) status.  ^5^ Five additional patients were enrolled, including 4 without biopsiable disease and one whose tissue was not evaluated using Exiqon’s miRCURY LNA microRNA Array (6^th^ gen).  Patient # for this miRNA analysis corresponds to patient number in the **Clinical Study as follows:** 1:**5**, 2:**6**, 3:**3**, 4:**1**, 5:**2**, 6:**7**, 7:**8**, 8:**9**, 9:**10**, 10:**11**, 11:**12**, 12:**15** | | | | | | | |
