# Supplementary material for: MicroRNAs induced in melanoma treated with combination targeted therapy of Temsirolimus and Bevacizumab
Source: J Transl Med. 2013 Sep 18;11:218. doi: 10.1186/1479-5876-11-218 (PMC3853033; doi:10.1186/1479-5876-11-218)
Supplement: Additional file 2 — Methods. Additional details of miRNA microarray analysis and qRT-PCR. [file 1479-5876-11-218-S2.doc]

**Additional File 2: Supplementary Data**

**Methods**

**Cells and Tissues.**

VMM18: VEGFR2+, BRAF^V600E^, NRAS^WT^,

VMM39: VEGFR2-, BRAF^WT^, NRAS^Q61L/R^

DM13: VEGFR2+, BRAF^V600E^ , NRAS^WT^

DMM122: VEGFR2-, BRAF^WT^, NRAS^WT^

**miRNA Microarray Analysis.** The hybridization was performed according to the miRCURY LNA microRNA Array instruction manual using a Tecan HS 4800 hybridization station (Tecan, Auctria). The microarray slides were then scanned using the Agilent G2565BA Microarray Scanner System (Agilent Technologies, Inc, USA) and the image analysis was performed using the ImaGene 9.0 software (BioDiscovery, Inc., USA).

**qRT-PCR.**

U6 small nuclear RNA, RNU6:

NCBI Accession #NR_004394, for Human, Mouse, and Rat

Forward and reverse primer sequences of ActinB and HPRT:

ActinB**:** GCTCCTCCTGAGCGCAAGT

CGTCATACTCCTGCTTGCTGAT

HPRT: TGGTCAAGGTCGCAAGCTT

GGGCATATCCTACAACAAACTTGTC.

Forward and reverse primers of 18 target genes:

AKT: ATCATTGCTTTCAGGGCTCTTG

TTACTCCAGAGAAGAAACTGTGTCTCA

LIN28B: GGCGGGCATGGCTGTAC

ACTTCGTGGAGGAAGCTTCTTG

CDK6: AGGAAAAATCTTGGACGTGATTG

TGGTTGGGCAGATTTTGAATG

PIK3C3: ACTGTGAAAAAGGTTCAGGATAAATTC

AAAGAGCATGGACACTCTCATCAA

MYC: GGCCCCCAAGGTAGTTATCC

TTTCCGCAACAAGTCCTCTTC

MYBL2: GCACCTGGAGGAGGACTTGA

CAGGCTTCCTCTTCTGCTTCTC

RASA1: TGGCCTACAAATACCACCATGA

GATGATATTGAACATCCGTGGATTC

DNMT3A: CGAGTCCAACCCTGTGATGAT

TGCCAACGGCCTGTTCATA

BCL2: GGAGGATTGTGGCCTTCTTTG

GTCATCCACAGGGCGATGTT

MCL1: GGCTGGGATGGGTTTGTG

CCAGCTCCTACTCCAGCAACA

CD276: AGCTGTGAGGAGGAGAATGCA

CTTCTTTGCTGTCAGAGTGTTTCAG

IGF1: CCCTCAAGCCTGCCAAGTC

CCTCTACTTGCGTTCTTCAAATGTAC

mTOR: CACTGGTCGGGACTTCTCTCA

GCACTGGCAGAGGTTTTCATG

SMARCA5: ATTGAAAGAGAAAACATGGAACTAGAAGA

GTGCGCCATCCATTTTACGT

SMARCD1: AGCGCCGAGCTGAGTTCTAC

GCTTGCTCTAATTCTTGTCGTCTCT

CCND1: CCCTGACGGCCGAGAAG

AGGTTCCACTTGAGCTTGTTCAC

HMGA2: CCGGTGAGCCCTCTCCTAAG

TCTCCAGTGGCTTCTGCTTTC

IGF-1R: ACTACATTGTGGGGAATAAGC

CGGGGGAATAACATTGCT
