# Supplementary figures and images for: MicroRNAs induced in melanoma treated with combination targeted therapy of Temsirolimus and Bevacizumab
Source: J Transl Med. 2013 Sep 18;11:218. doi: 10.1186/1479-5876-11-218 (PMC3853033; doi:10.1186/1479-5876-11-218)

## Slide 1
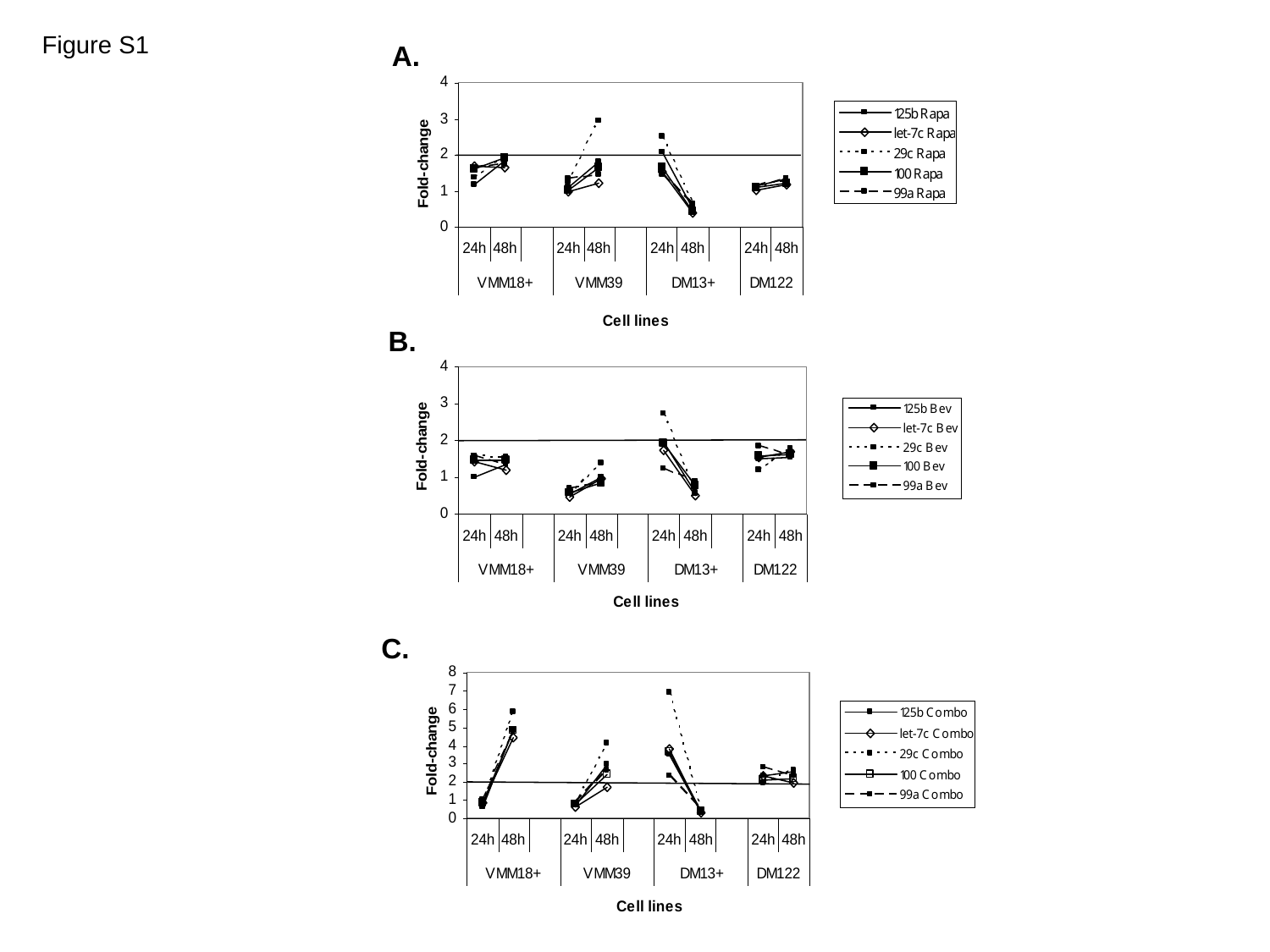

Figure S1
A.
B.
C.

Supplement: Additional file 3: Figure S1 — In vitro analysis: Part I. Expression of the first set of miRNAs (miR-125b, let-7c, -29c, -100, -99a) in four melanoma cell lines after culturing with media alone, rapamycin (Temsirolimus analogue), Bevacizumab, or combination of rapamycin and Bevacizumab. [file 1479-5876-11-218-S3.ppt]

## Slide 1
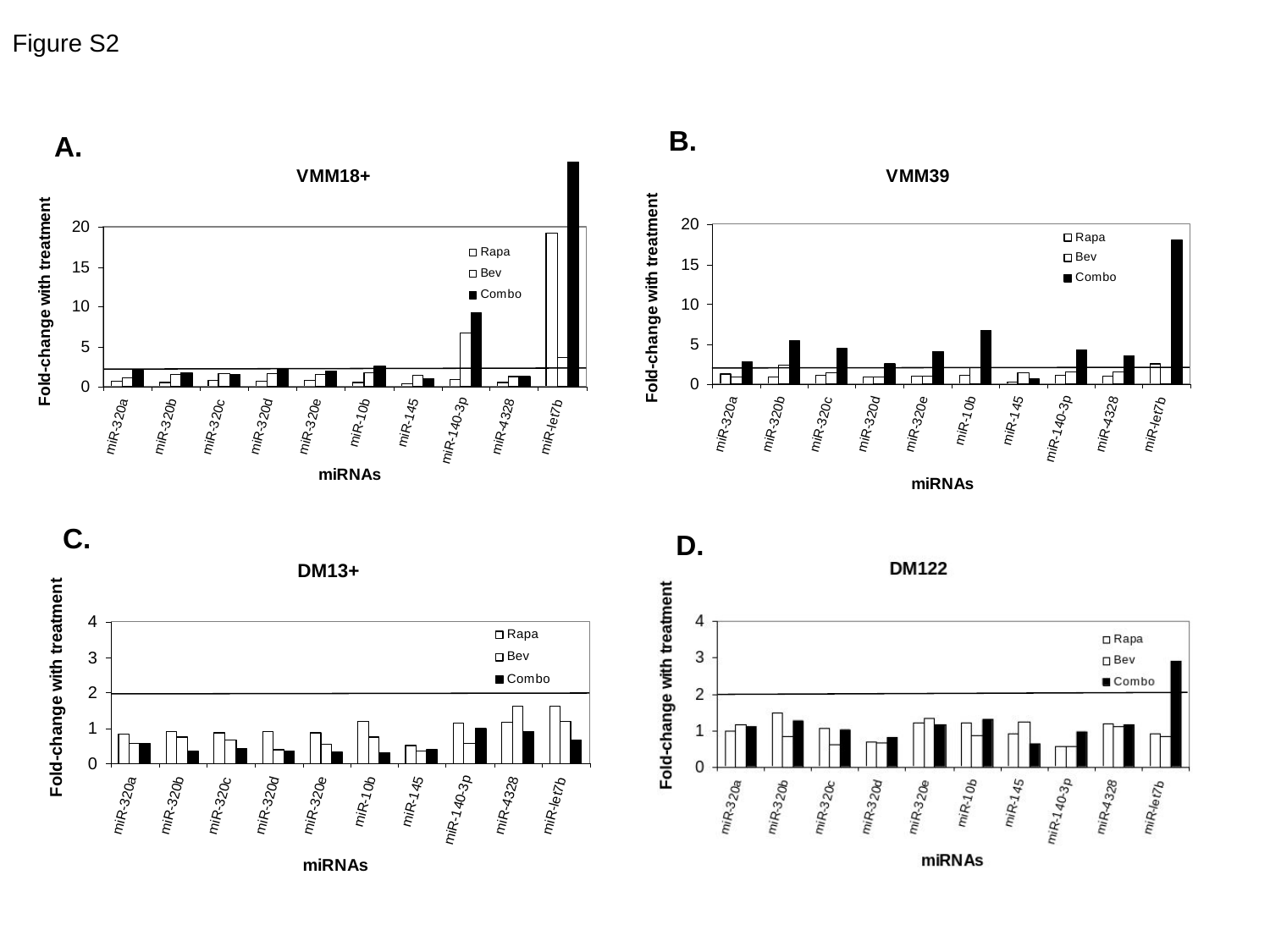

Figure S2
B.
A.
*
C.
D.

Supplement: Additional file 4: Figure S2 — In vitro analysis: Part II. Analysis of the remaining 10 miRNAs. [file 1479-5876-11-218-S4.ppt]
