# Supplementary material for: MicroRNAs induced in melanoma treated with combination targeted therapy of Temsirolimus and Bevacizumab
Source: J Transl Med. 2013 Sep 18;11:218. doi: 10.1186/1479-5876-11-218 (PMC3853033; doi:10.1186/1479-5876-11-218)
Supplement: Additional file 5: Table S2 — Correlation between changes in miRNA and mRNA with combination treatment: correlation coefficients and p-values. Negative correlations and significant p-values are in bold. [file 1479-5876-11-218-S5.docx]

**Table S2.** Correlation between changes in miRNA and mRNA with combination treatment: correlation coefficients and *p*-values. Negative correlations and significant p-values are in bold.

| **Pair** | **Correlation**  **Coefficient** | **p-value** |
| --- | --- | --- |
| Let-7b-LIN28B | **-0.9318** | **0.0008** |
| Let-7c-LIN28B | **-0.9195** | **0.0012** |
| Let-7b-HMGA2 | **-0.7039** | 0.0514 |
| Let-7c-C-MYC | **-0.5852** | 0.1275 |
| 99a-SMARCA5 | **-0.5655** | 0.1440 |
| Let-7b-C-MYC | **-0.5045** | 0.2023 |
| 125b-LIN28B | **-0.4259** | 0.2927 |
| 320-CDK6 | **-0.3891** | 0.3407 |
| 29c-BCL2 | **-0.3479** | 0.3984 |
| 320- PI3-K | **-0.3076** | 0.4586 |
| 29c-MCL1 | **-0.2894** | 0.4869 |
| Let-7b-CYCLIND1 | **-0.2008** | 0.6334 |
| 29c-PI3-K | **-0.1342** | 0.7514 |
| Let-7c-RAS | **-0.0363** | 0.9320 |
| 29c-B-MYB | **-0.0065** | 0.9878 |
| 145-C-MYC | 0.0000 | 1.0000 |
| 100-mTOR | 0.0000 | 1.0000 |
| 100-SMARCA5 | 0.0000 | 1.0000 |
| 100-SMARCD1 | 0.0000 | 1.0000 |
| Let-7b-RAS | 0.0208 | 0.9611 |
| 99a-SMARCD1 | 0.1516 | 0.7202 |
| 29c-CDK6 | 0.1963 | 0.6412 |
| 99a-mTOR | 0.2345 | 0.5761 |
| 125b-AKT | 0.2648 | 0.5262 |
| 29c-DNMT3A/3B | 0.4958 | 0.2115 |
